# Prediction of postoperative complications after hepatectomy with dynamic monitoring of central venous oxygen saturation

**DOI:** 10.1186/s12893-023-02238-6

**Published:** 2023-11-14

**Authors:** Shinya Ida, Yoshifumi Morita, Akio Matsumoto, Ryuta Muraki, Ryo Kitajima, Satoru Furuhashi, Makoto Takeda, Hirotoshi Kikuchi, Yoshihiro Hiramatsu, Hiroya Takeuchi

**Affiliations:** 1 Department of Surgery, Hamamatsu University School of Medicine, 1-20-1 Handayama, Hamamatsu, 431-3192 Japan; 2 Division of Surgical Care, Morimachi, Hamamatsu University School of Medicine, Hamamatsu, Japan; 3 Department of Perioperative Functioning Care and Support, Hamamatsu University School of Medicine, Hamamatsu, Japan

**Keywords:** Flo Trac System, Central venous oxygen saturation, Hepatectomy, Comprehensive complication index, Anesthetic management

## Abstract

**Background:**

The usefulness of static monitoring using central venous pressure has been reported for anesthetic management in hepatectomy. It is unclear whether intra-hepatectomy dynamic monitoring can predict the postoperative course. We aimed to investigate the association between intraoperative dynamic monitoring and post-hepatectomy complications. Furthermore, we propose a novel anesthetic management strategy to reduce postoperative complication.

**Methods:**

From 2018 to 2021, 93 patients underwent hepatectomy at our hospital. Fifty-three patients who underwent dynamic monitoring during hepatectomy were enrolled. Flo Trac system was used for dynamic monitoring. The baseline central venous oxygen saturation (ScvO_2_) was defined as the average ScvO_2_ for 30 min after anesthesia induction. ScvO_2_ fluctuation (ΔScvO_2_) was defined as the difference between the baseline and minimum ScvO_2_. Postoperative complications were evaluated using the comprehensive complication index (CCI).

**Results:**

Patients with ΔScvO_2_ ≥ 10% had significantly higher CCI scores (0 vs. 20.9: *p* = 0.043). In univariate analysis, patients with higher CCI scores demonstrated significantly higher preoperative C-reactive protein-to-lymphocyte ratio (7.51 vs. 24.49: *p* = 0.039), intraoperative bleeding (105 vs. 581 ml: *p* = 0.008), number of patients with major hepatectomy (4/45 vs. 3/8: *p* = 0.028), and number of patients with ΔScvO_2_ ≥ 10% (11/45 vs. 6/8; *p* = 0.010). Multivariate logistic regression analysis revealed that ΔScvO_2_ ≥ 10% (odds ratio: 9.53, *p* = 0.016) was the only independent predictor of elevated CCI.

**Conclusions:**

Central venous oxygen saturation fluctuation during hepatectomy is a predictor of postoperative complications. Anesthetic management based on intraoperative dynamic monitoring and minimizing the change in ScvO_2_ is a potential strategy for decreasing the risk of post-hepatectomy complications.

**Supplementary Information:**

The online version contains supplementary material available at 10.1186/s12893-023-02238-6.

## Background

Post-hepatectomy complications have decreased due to technological advances and improved perioperative management. However, post-hepatectomy liver failure, a serious complication, still occurs in 1.2% to 32% of patients after hepatectomy [1, 2]. The occurrence of post-hepatectomy complications is partially related to intraoperative bleeding and perioperative blood transfusion. During hepatectomy, blood loss can be minimized using the Pringle maneuver and low central venous pressure (CVP) management. Maintaining the CVP < 5 cmH_2_O during hepatectomy reportedly reduces intraoperative bleeding and postoperative complications [3, 4].

CVP measurement involves a static fluid monitoring system; thus, the CVP may not adequately reflect intraoperative fluid volume and tissue oxygen demand. Recently, the Flo Trac system (FTS) has attracted attention as a dynamic fluid monitoring system. The FTS can measure multiple fluid indicators every 20 s, allowing for rapid fluid volume adjustments during surgery [5–7]. Among the FTS parameters, intraoperative central venous oxygen saturation (ScvO_2_) fluctuation (ΔScvO_2_) is an indicator of increased total bilirubin level after hepatectomy [5].

Although the FTS is reported useful for appropriate intraoperative anesthetic management [5, 7, 8], no study has reported an association between ScvO_2_ and postoperative complications. The hypothesis of this study is that intraoperative dynamic monitoring will reveal predictors of postoperative complications in hepatectomy. Finally, we propose a novel anesthetic management strategy to reduce the occurrence of postoperative complications.

## Methods

In this retrospective cohort study, we enrolled patients who underwent hepatectomy with FTS-monitored anesthetic management in our institution from August 2018 to December 2021. Informed consent for data collection was obtained in the form of an opt-out on the institution website. This study was approved by the ethics review board of our institution (approval number 17–124) in accordance with the ethical guidelines of the Japanese Ministry of Health, Labour, and Welfare regarding clinical studies.

### Surgical indication and intraoperative procedures

The extent of hepatectomy was determined based on the primary disease as well as the number and localization of tumors. A major hepatectomy was defined as the removal of one or more segments of the liver. A minor hepatectomy was defined as the removal of less than one segment of the liver. Preoperatively, the indocyanine green test was performed to evaluate the liver function. In patients who underwent major hepatectomy, the remnant K value (remnant liver volume multiplied by indocyanine green disappearance rate) was confirmed to be at least 0.05. Hepatic transection was mainly performed using the Cavitron ultrasonic surgical aspirator (Valleylab, Boulder, CO, USA) and ultrasonic scalpels, with an intermittent application of the Pringle maneuver, which involves clamping the portal triad for 15 and 10 min in patients with normal liver and liver dysfunction, respectively, and releasing the clamp at 5-min intervals. A hemostatic device on the cutting liver surface used saline-coupled soft coagulation of an IO advanced monopolar electrode with a VIO 300 D system (Erbe Elektromedizin GmbH, Tübingen, Germany).

### Intraoperative anesthetic management

**Fig. 1:**
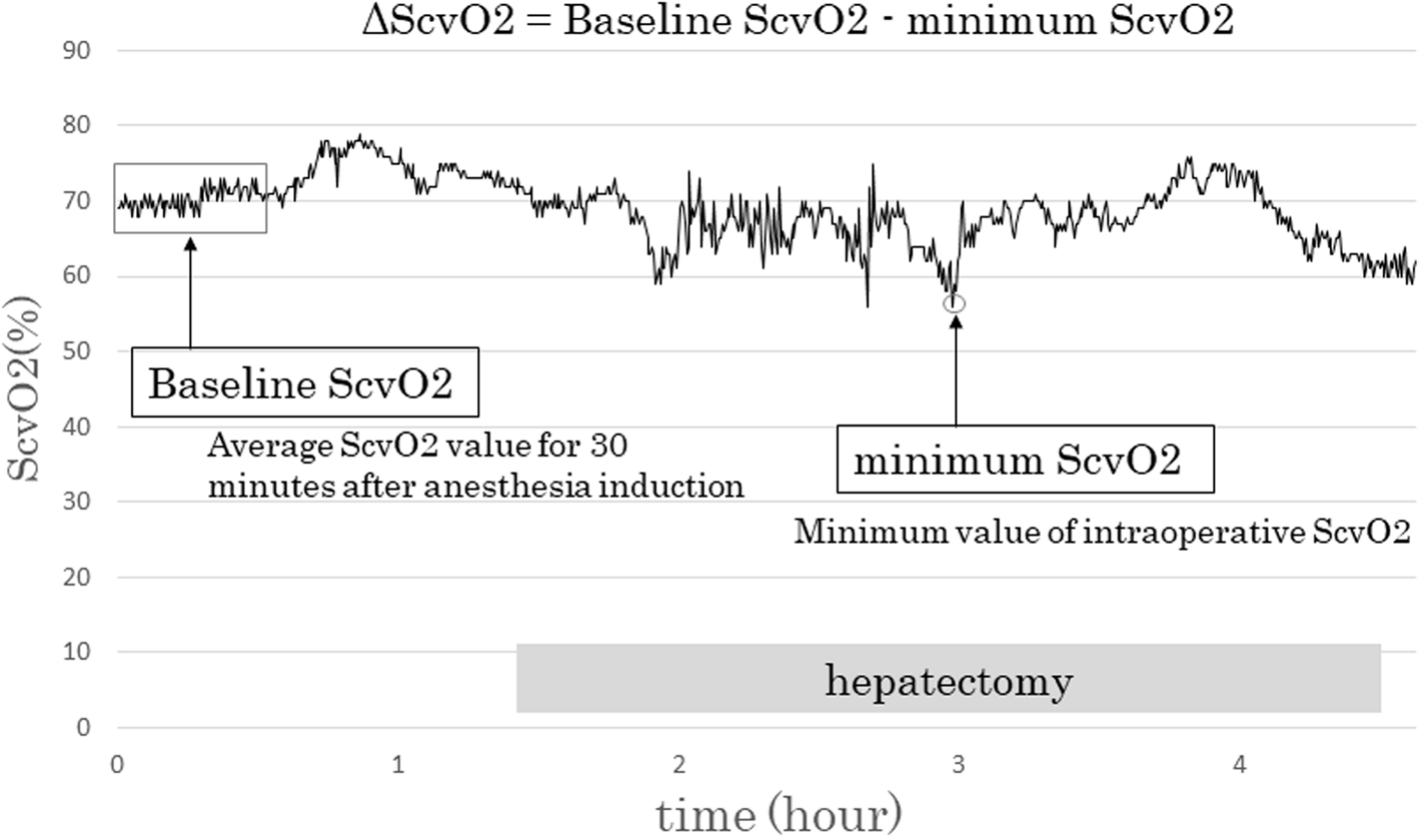
Definitions of baseline and minimum central venous oxygen saturation (ScvO_2_). The baseline and minimum ScvO_2_ values are defined as the average ScvO_2_ value for 30 min after anesthesia induction and the lowest intraoperative ScvO_2_ value, respectively. ΔScvO_2_ is defined as the difference between the baseline and minimum ScvO_2_ values

Each anesthesiologist determined the infusion fluid volume and ventilator settings. During anesthesia, data were collected using a dedicated transducer (FloTrac, Edwards Lifesciences) connected to the radial arterial line and a Vigileo™ monitor (Edwards Lifesciences) or EV1000 Critical Care monitor (Edwards Lifesciences) for continuous monitoring. This monitoring strategy analyzes the pressure waveform 20 times per second for 100 s, captures 2,000 data points for analysis, and performs calculations on the data acquired during the last 20 s. A PreSep central venous oximetry catheter (Edwards Lifesciences) was used to facilitate continuous ScvO_2_ monitoring [9]. The catheter tip was inserted into the superior vena cava and emitted near-infrared rays, which allowed for continuous blood oxygen saturation measurement. The radial arterial line was connected to the Vigileo™ monitor or EV1000 Critical Care monitor to allow for stroke volume variation (SVV) measurement. The SVV represents the respiratory variability in stroke volume and is affected by the vascular compliance and peripheral resistance. The vascular compliance is estimated from nomograms based on age, sex, height, and weight, whereas the peripheral resistance is determined using radial artery waveforms [10, 11]. In this study, the baseline ScvO_2_ and ΔScvO_2_ were defined with a simple modification of previously reported method [5]. The baseline ScvO_2_ was defined as the average ScvO_2_ value for 30 min after anesthesia induction. The minimum ScvO2 was defined as the lowest intraoperative ScvO2 value. ΔScvO_2_ was defined as the difference between the baseline and minimum ScvO_2_ values (Fig. 1). Moreover, the baseline SVV was defined as the average SVV value for 30 min after anesthesia induction. The maximum SVV was defined as the highest intraoperative SVV value. SVV fluctuation (ΔSVV) was defined as the difference between the baseline and maximum SVV values.

### Classification of postoperative complications

Postoperative complications were classified according to the Clavien-Dindo grading system [12] and evaluated using the comprehensive complication index (CCI), which is a score obtained by weighing all postoperative complications based on their severity [13].

### Statistical analysis

Continuous variables were expressed as median (interquartile range) and compared using the Mann–Whitney *U*-test or Student's *t*-test. Pearson's chi-square test or Fisher's exact test was used to compare categorical variables. A multivariate logistic regression analysis was performed to identify predictors of postoperative complications. Odds ratios (ORs) and 95% confidence intervals (CIs) were calculated. Statistical analysis was performed using SPSS version 26 (IBM Corp., Armonk, NY, USA), and *p*-values < 0.05 were considered statistically significant.

## Results

**Fig. 2:**
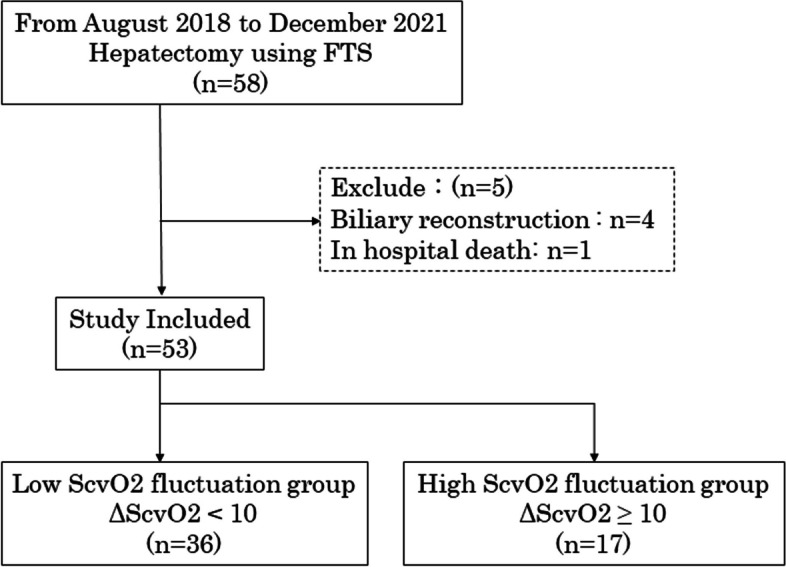
Study flow chart. From August 2018 to December 2021, 58 patients underwent hepatectomy using Flo Trac system (FTS)-monitored anesthetic management. Four and one patients with biliary reconstruction and postoperative death were excluded, respectively. Hence, the 53 included patients were divided into two groups: low (central venous oxygen saturation fluctuation [ΔScvO_2_] < 10%, *n* = 36) and high (ΔScvO_2_ ≥ 10%, *n* = 17) ScvO_2_ groups. ΔScvO_2_ is defined as the difference between the baseline and minimum ScvO2 values

Of 93 patients who underwent hepatectomy during the study period at our institute, 58 patients were received anesthetic management with FTS monitoring. We excluded four patients with biliary reconstruction and one patient with aspiration pneumonia-induced in-hospital death. Therefore, 53 patients were enrolled in this study (Fig. 2).

### Basic patient characteristics

Forty patients were men. All patients had liver diseases with Child–Pugh and liver damage classifications of A or B. One patient had a history of atrial fibrillation (Table 1).

**Table 1:** Baseline patient characteristics

	All patients (*n* = 53)
Age	70 (63–76)
Sex (male/female)	40: 13
BMI	22.3 (20.5–24.7)
ASA (1: 2: 3)	1: 48: 4
Hypertension (%)	15/53 (28.3)
Hyperlipidemia (%)	3/53 (5.7)
Diabetes (%)	12/53 (22.6)
Atrial fibrillation (%)	1/53 (1.9)
Primary disease (HCC: others)	29: 24
PTPE (%)	4/53 (7.5)
History of hepatitis virus infection (HBV: HCV: no)	6: 7: 40
Child–Pugh classification (A: B: C)	52: 1: 0
Liver damage classification (A: B: C)	46: 7: 0

*BMI* body mass index, *ASA* American Society of Anesthesiologists, *HCC* hepatocellular carcinoma, *PTPE* Percutaneous transhepatic portal vein embolization, *HCV* hepatitis C virus, *HBV* hepatitis B virus

### Patient characteristics stratified by ΔScvO_2_ and average SVV

A previous study reported that ΔScvO_2_ ≥ 10.2% was a significant predictor of postoperative liver dysfunction. Therefore, the 53 patients were divided into two groups: low (ΔScvO_2_ < 10%, *n* = 36) and high (ΔScvO_2_ ≥ 10%, *n* = 17) ΔScvO_2_ groups (Fig. 2). There was no significant difference in patient background and preoperative clinicopathological factors between the two groups (Table 2). A previous study reported that intraoperative average SVV ≥ 13.6 was a significant predictor of postoperative liver dysfunction [5]. Hence, the 53 patients were divided into two groups: low (SVV < 13.6, *n* = 45) and high (SVV ≥ 13.6, *n* = 8) SVV groups. Additional file 1 compares the patient background, preoperative treatment, and preoperative blood test findings between the two groups. There were significant between-group differences in the levels of total bilirubin (0.7 [0.6–1.0] vs. 1.2 [0.9–1.5] mg/dL; *p* = 0.019), alkaline phosphatase (239 [199–310] vs. 202 [159–205] IU/l; *p* = 0.021), γ-glutamyltranspeptidase (55 [27–92] vs. 27 [14–27] IU/l; *p* = 0.026), and C-reactive protein (0.13 [0.05–0.37] vs. 0.05 [0.03–0.09] mg/dL; *p* = 0.028) as well as C-reactive protein-to-lymphocyte ratio (CLR) (9.87 [4.88–32.42] vs. 4.49 [2.68–8.36]; *p* = 0.034) and C-reactive protein-to-albumin ratio (0.030 [0.011–0.097] vs. 0.010 [0.007–0.021]; *p* = 0.028).

**Table 2:** Patient data stratified by ΔScvO2

	Low ScvO2 fluctuation (ΔScvO2 < 10%) (*n* = 36)	High ScvO_2_ fluctuation (ΔScvO2 ≥ 10%) (*n* = 17)	*p*
**Preoperative factors**			
Age	70 (60–77)	69 (66–74)	0.742
Sex (male/female)	27: 9	13: 4	0.597
BMI	22.3 (20.5–24.6)	22.4 (19.9–25.2)	0.874
ASA (1: 2: 3)	1: 31: 4	0: 17: 0	0.382
Hypertension (%)	11/36 (30.6)	4/17 (23.5)	0.426
Hyperlipidemia (%)	3/36 (8.3)	0/17 (0)	0.305
Diabetes (%)	7/36 (19.4)	5/17 (29.4)	0.318
Primary disease (HCC: others)	23: 13	6: 11	0.051
PTPE (%)	1/36 (2.8)	3/17 (17.6)	0.092
History of hepatitis virus infection (HBV: HCV: no)	5: 4: 27	1: 3: 13	0.597
Child–Pugh classification (A: B: C)	35: 1: 0	17: 0: 0	0.679
Liver damage classification (A: B: C)	30: 6: 0	16: 1: 0	0.269
White blood cell (/μL)	5260 (4405–6193)	5450 (4460–6560)	0.804
Platelet (× 10^4^/μL)	18.6 (14.6–24.0)	22.2 (17.5–23.7)	0.423
Prothrombin activity (%)	103 (97–113)	101 (94–115)	0.600
Aspartate transaminase (IU/L)	28 (22–40)	24 (22–25)	0.088
Alanine transaminase (IU/L)	23 (15–33)	19 (16–23)	0.340
Total bilirubin (mg/dL)	0.7 (0.6–1.1)	0.9 (0.5–1.2)	0.625
Alkaline phosphatase (IU/L)	226 (201–289)	230 (182–263)	0.542
γ-Glutamyltranspeptidase (IU/L)	45 (25–89)	45 (25–64)	0.790
Albumin (g/dL)	4.2 (3.9–4.4)	4.2 (4.0–4.4)	0.863
Cholinesterase (IU/L)	279 (240–322)	260 (224–289)	0.607
Total cholesterol (mg/dL)	193 (162–217)	177 (148–200)	0.261
C-reactive protein (mg/dL)	0.09 (0.04–0.25)	0.16 (0.05–0.65)	0.417
HbA1c (%)	6.1 (5.5–6.5)	5.9 (5.6–6.0)	0.498
ICG R15 (%)	18.1 (12.0–24.6)	13.3 (11.3–16.5)	0.091
NLR	2.85 (1.75–4.08)	3.70 (2.43–4.15)	0.073
PLR	135.3 (108.8–235.2)	193.8 (146.6–254.1)	0.086
LMR	3.43 (2.55–4.32)	3.04 (2.53–4.97)	0.844
CLR	8.33 (4.20–17.96)	11.85 (5.07–82.25)	0.303
CAR	0.022 (0.010–0.059)	0.039 (0.013–0.151)	0.423
**Intraoperative factors**			
Operative method (minor: major)	34: 2	12: 5	**0.017**
laparotomy: laparoscopy	15: 21	13: 4	**0.018**
Operation time (min)	294 (253–350)	301 (261–397)	0.317
Intraoperative bleeding (mL)	105 (56–383)	435 (50–660)	0.093
Urine volume (mL)	255 (110–398)	250 (140–372)	0.985
Transfusion (%)	3/36 (8.3)	2/17 (11.8)	0.520
Crystalloid fluid volume (mL)	1850 (1450–2585)	2200 (1850–2800)	0.185
Intraoperative in–out balance (mL/kg/h)	6.83 (5.24–8.37)	6.56 (5.77–7.10)	0.939
Total Pringle maneuver time (min)	90 (60–115)	75 (56–90)	0.175
Hepatectomy time (min)	118 (91–164)	109 (76–154)	0.667
**Postoperative factors**			
Max white blood cell (/μL)	10,780 (8440–12410)	8970 (7130–11010)	0.148
Min platelet (× 10^4^/μL)	13.1 (9.4–17.0)	14.7 (12.0–17.0)	0.640
Min prothrombin activity (%)	71 (61–84)	66 (53–74)	0.114
Max aspartate transaminase (IU/L)	210 (152–404)	358 (224–597)	0.072
Max alanine transaminase (IU/L)	206 (123–378)	350 (228–467)	0.057
Max total bilirubin (mg/dL)	1.2 (1.0–1.7)	1.7 (1.2–2.1)	0.079
Min albumin (g/dL)	3.1 (2.8–3.3)	2.8 (2.7–3.0)	0.092
Min cholinesterase (IU/L)	179 (160–216)	160 (124–172)	**0.036**
Max C-reactive protein (mg/dL)	8.40 (5.94–11.94)	9.47 (7.85–12.39)	0.542
Complications of CDC grade IIIa or higher (%)	0/36 (0)	2/17 (11.8)	0.099
CCI	0 (0–14.4)	20.9 (0–24.2)	**0.043**
Postoperative length of stay (day)	9 (8–12)	11 (9–14)	0.135
Surgical site infection (%)	3/36 (8.3)	2/17 (11.8)	0.520
Bile leakage (%)	0/36 (0)	1/17 (5.9)	0.321
Pleural effusion (%)	3/36 (8.3)	5/17 (29.4)	0.059
Ascites (%)	4/36 (11.1)	3/17 (17.6)	0.398
Pneumonia (%)	0/36 (0)	1/17 (5.9)	0.321
Diarrhea (%)	2/36 (5.6)	1/17 (5.9)	0.695
Delayed gastric emptying (%)	0/36 (0)	2/17 (11.8)	0.099

Continuous data are presented as median (interquartile range), whereas categorical data are shown as number of patients. Significant *p*-values are in boldface

*ScvO2* central venous oxygen saturation, *BMI* body mass index, *ASA* American Society of Anesthesiologists, *HCC* hepatocellular carcinoma, *PTPE* Percutaneous transhepatic portal vein embolization, *HCV* hepatitis C virus, *HBV* hepatitis B virus, *HbA1c* Hemoglobin A1c, *ICGR15* indocyanine green retention rate at 15 min, *NLR* neutrophil-to-lymphocyte ratio, *PLR* platelet-to-lymphocyte ratio, *LMR* lymphocyte-to-monocyte ratio, *CLR* C-reactive protein-to-lymphocyte ratio, *CAR* C-reactive protein-to-albumin ratio, *CDC* Clavien-Dindo classification, *CCI* Comprehensive complication index

### Intraoperative factors stratified by ΔScvO_2_ and average SVV

Table 2 also shows the intraoperative factors stratified by ΔScvO2. There was a significant between-group difference in the number of patients who underwent major (2/36 vs. 5/17; *p* = 0.017) and laparotomy (15/36 vs. 13/17; *p* = 0.018) hepatectomy. Operation time, intraoperative bleeding, intraoperative fluid volume, and hepatectomy time were not significantly different between the two groups. Additional file 1 also presents intraoperative factors stratified by average SVV. Intraoperative bleeding (275 [85–542] vs. 33 [13–86] ml; *p* = 0.005) was significantly different between the two groups.

### Postoperative course stratified by ΔScvO_2_ and average SVV

Table 2 also shows postoperative course and details of postoperative complications stratified by ΔScvO2. The minimum cholinesterase level (179 [160–216] vs. 160 [124–172] IU/l; *p* = 0.036) and CCI score (0 [0–14.4] vs. 20.9 [0–24.2]; *p* = 0.043) were significantly different between the two groups. There was no significant between-group difference in the incidence of complications with Clavien-Dindo grade III or more. High ΔScvO2 tended to associate with more frequent pleural effusion and delayed gastric emptying. There was no patient of post hepatectomy liver failure. Additional file 1 also shows postoperative blood test findings and postoperative course stratified by average SVV. There was no significant between-group difference in the incidence of complications with Clavien-Dindo grade III or more and CCI score.

### Patient characteristics stratified by CCI score

The abovementioned results suggested that the intraoperative ΔScvO_2_ was related to postoperative complication occurrence. Although the cut off value of the CCI is considered to be different depending on each surgical procedures, the median CCI score for the study participants was 20.9; hence, the participants were divided into two groups: low (CCI < 21, *n* = 45) and high (CCI ≥ 21, *n* = 8) groups.

Table 3 presents patient characteristics stratified by CCI score. We found no significant between-group difference in patient background and preoperative clinicopathological factors except for CLR (7.51 [4.02–16.39] vs. 24.49 [9.83–101.19]; *p* = 0.039) in univariate analysis.

**Table 3:** Patient characteristics stratified by CCI

	Low CCI (CCI < 21) (*n* = 45)	High CCI (CCI ≥ 21) (*n* = 8)	*p*
Age	70 (61–75)	70 (68–76)	0.742
Sex (male: female)	32: 13	8: 0	0.087
BMI	22.30 (20.50–24.68)	22.15 (20.67–23.49)	0.874
ASA (1: 2: 3)	1: 40: 4	0: 8: 0	1.000
Hypertension (%)	11/45 (24.4)	4/8 (50.0)	0.147
Hyperlipidemia (%)	2/45 (4.5)	1/8 (12.5)	0.394
Diabetes (%)	10/45 (22.2)	2/8 (25.0)	0.588
Primary disease (HCC: others)	24: 21	5: 3	0.466
PTPE (%)	2/45 (4.5)	2/8 (25.0)	0.104
History of hepatitis virus (HBV: HCV: no)	5: 4: 36	1: 3: 4	0.090
Child–Pugh classification (A: B: C)	44: 1: 0	8: 0: 0	0.849
Liver damage classification (A: B: C)	39: 6: 0	7: 1: 0	0.717
White blood cell (/μL)	5340 (4460–6560)	5005 (4285–5928)	0.533
Platelet (× 10^4^/μL)	19.6 (16.5–24.4)	17.7 (14.7–19.7)	0.275
Prothrombin activity (%)	103 (97–115)	97 (95–105)	0.346
Aspartate transaminase (IU/L)	25 (22–40)	25 (20–33)	0.617
Alanine transaminase (IU/L)	22 (15–32)	18 (15–21)	0.371
Total bilirubin (mg/dL)	0.7 (0.6–1.1)	0.9 (0.6–1.1)	0.874
Alkaline phosphatase (IU/L)	230 (202–285)	202 (182–259)	0.398
γ-Glutamyltranspeptidase (IU/L)	41 (23–66)	52 (26–113)	0.471
Albumin (g/dL)	4.2 (3.9–4.4)	4.1 (3.6–4.2)	0.233
Cholinesterase (IU/L)	278 (240–324)	260 (221–285)	0.486
Total cholesterol (mg/dL)	190 (155–214)	196 (177–202)	0.583
C-reactive protein (mg/dL)	0.09 (0.04–0.22)	0.43 (0.08–0.74)	0.128
HbA1c (%)	6.0 (5.5–6.5)	5.9 (5.5–5.9)	0.456
ICG R15 (%)	15.5 (11.3–22.5)	16.7 (13.2–26.7)	0.243
NLR	2.96 (2.04–3.90)	3.86 (2.89–4.41)	0.233
PLR	157.5 (116.0–254.1)	202.9 (140.7–243.8)	0.441
LMR	3.43 (2.67–4.76)	2.71 (2.51–3.98)	0.512
CLR	7.51 (4.02–16.39)	24.49 (9.83–101.19)	**0.039**
CAR	0.023 (0.009–0.056)	0.100 (0.019–0.213)	0.142

Continuous data are presented as median (interquartile range), whereas categorical data are shown as number of patients. Significant *p*-values are in boldface

*CCI* Comprehensive complication index, *BMI* body mass index, *ASA* American Society of Anesthesiologists, *HCC* hepatocellular carcinoma, *PTPE* Percutaneous transhepatic portal vein embolization, *HCV* hepatitis C virus, *HBV* hepatitis B virus, *HbA1c* Hemoglobin A1c, *ICGR15* indocyanine green retention rate at 15 min, *NLR* neutrophil-to-lymphocyte ratio, *PLR* platelet-to-lymphocyte ratio, *LMR* lymphocyte-to-monocyte ratio, *CLR* C-reactive protein-to-lymphocyte ratio, *CAR* C-reactive protein-to-albumin ratio

### Intraoperative factors stratified by CCI score

There were significant differences in the number of patients who underwent major hepatectomy (4/45 vs. 3/8; *p* = 0.028) and in intraoperative bleeding (105 [35–382] vs. 581 [465–694] ml; *p* = 0.008) (Table 4) in univariate analysis.

**Table 4:** Intraoperative factors stratified by CCI

	Low CCI (CCI < 21) (*n* = 45)	High CCI (CCI ≥ 21) (*n* = 8)	*p*
Operative method (minor: major)	41: 4	5: 3	**0.028**
laparotomy: laparoscopy	22: 23	6: 2	0.164
Operation time (min)	293 (247–349)	366 (287–397)	0.135
Intraoperative bleeding (mL)	105 (35–382)	581 (465–694)	**0.008**
Urine volume (mL)	279 (110–395)	178 (122–279)	0.371
Transfusion (%)	5/45 (11.1)	0/8 (0)	0.426
Crystalloid fluid volume (mL)	1850 (1450–2580)	2550 (1850–2775)	0.105
Intraoperative in–out balance (mL/kg/h)	6.92 (5.59–8.30)	5.83 (5.42–6.26)	0.243
Lymph node dissection (%)	1 (2.2)	1 (12.5)	0.282
Total Pringle maneuver time (min)	90 (70–120)	60 (55–103)	0.219
Hepatectomy time (min)	116 (83–165)	130 (79–172)	0.909

Continuous data are presented as median (interquartile range), whereas categorical data are shown as number of patients. Significant *p*-values are in boldface

*CCI* Comprehensive complication index

### FTS measurements stratified by CCI score

We observed a significant between-group difference in the number of patients with ΔScvO_2_ ≥ 10% (11/45 vs. 6/8; *p* = 0.010) (Table 5) in univariate analysis. However, the average SVV, maximum SVV, and ΔSVV were not significantly different between the two groups. Furthermore, the pre- and postoperative CVP as well as the maximum intraoperative CVP were not significantly different between the two groups. Lactate levels measured immediately after surgery were not significantly different between the two groups.

**Table 5:** FTS measurements

	Low CCI (CCI < 21) (*n* = 45)	High CCI (CCI ≥ 21) (*n* = 8)	*p*
Average ScvO_2_	80.0 (72.3–83.2)	75.2 (70.9–79.1)	0.214
Minimum ScvO_2_	71 (62–76)	61 (56–69)	0.114
ΔScvO_2_	6.3 (5.3–10.0)	13.1 (8.9–20.8)	0.135
10 ≤ (%)	11/45 (24.4)	6/8 (75.0)	**0.010**
Average SVV	9.4 (7.4–12.1)	8.8 (7.9–10.2)	0.652
Maximum SVV	22 (17–27)	21 (17–24)	0.687
ΔSVV	11.98 (8.50–19.04)	13.09 (10.28–17.04)	0.932
CVP at start of surgery	5 (3–8)	6 (4–8)	0.720
CVP at end of surgery	6 (4–8)	5 (3–10)	0.801
Maximum CVP	11 (8–14)	11 (9–13)	0.968
Lactate level value immediately after surgery (mg/dL)	2.0 (1.4–2.7)	2.2 (1.8–3.1)	0.450

Continuous data are presented as median (interquartile range), whereas categorical data are shown as number of patients. Significant *p*-values are in boldface

*CCI* Comprehensive complication index, *ScvO*_*2*_ central venous oxygen saturation, *ΔScvO*_*2*_ central venous oxygen saturation fluctuation, *SVV* stroke volume variation, *ΔSVV* stroke volume variation fluctuation, *CVP* central venous pressure

### ΔScvO2 was an independent predictor of higher CCI scores

Multivariate logistic regression analysis revealed the discriminative capacity of high CCI scores (Table 6). The CLR (median, 9.7), intraoperative bleeding (median, 240 mL), the number of cases with major hepatectomy and ΔScvO2 ≥ 10% were included in the multivariate analysis. The result revealed that ΔScvO2 ≥ 10% (p = 0.016, odds ratio: 9.53) was the only independent predictor of higher CCI scores.

**Table 6:** Multivariate analysis

	Low CCI (CCI < 21) (*n* = 45)	High CCI (CCI ≥ 21) (*n* = 8)	Multivariate analysis *p*-value	Odds ratio	95% CI
CLR (< 9.7: 9.7 ≤)	25: 20	1: 7			
Operative method (< H1: H2 ≤)	41: 4	5: 3			
Intraoperative bleeding (mL) (< 240: 240 ≤)	25: 20	1: 7			
ΔScvO_2_ (< 10: 10 ≤)	34: 11	2: 6	**0.016**	9.53	1.523–59.655

Continuous data are presented as median (interquartile range), whereas categorical data are shown as number of patients. Significant *p*-values are in boldface

*CCI* Comprehensive complication index, *CLR* C-reactive protein-to-lymphocyte ratio, *ΔScvO*_*2*_ central venous oxygen saturation fluctuation

## Discussion

This study evaluated the intraoperative ScvO_2_ and SVV measured using the FTS in patients undergoing hepatectomy. ΔScvO_2_ showed a significant positive correlation with CCI score, whereas, average SVV, maximum SVV, and ΔSVV were not significantly correlated with CCI score. Multivariate analysis identified ΔScvO_2_ as an independent predictor of elevated CCI scores.

Although recent studies have reported that the mortality rate of patients undergoing hepatectomy is less than 5%, post-hepatectomy complication rates range from 20 to 40%, depending on the extent of resection and liver function [14, 15]. Intraoperative bleeding constitutes a major factor affecting post-hepatectomy outcomes [16, 17]. Intermittent blockage of hepatic blood flow using the Pringle maneuver can reduce intraoperative bleeding; nevertheless, it causes hepatocyte ischemia and reperfusion, leading to liver injury and elevated serum lactate levels [18, 19]. Patients with elevated lactate levels immediately after hepatectomy have a higher risk of postoperative morbidity and mortality [20]. In this study, there was no relationship between the CCI score and postoperative lactate levels. Postoperative lactate level may not be a good predictor of complications in patients undergoing minimally invasive surgery and minor hepatectomy.

Generally, lowering the CVP during hepatectomy reduces hepatic venous and sinusoidal pressures, thereby minimizing bleeding from the liver parenchyma [16, 21]. During hepatectomy, it is recommended to maintain the CVP < 5 cmH_2_O [3, 22, 23]. However, the CVP is affected by the patient's position during surgery, intrathoracic pressure, and operator compression or clamping of the inferior vena cava, hepatic vein, and portal vein [24]. Furthermore, the CVP is a static hemodynamic monitoring indicator, and thus it is inaccurate for diagnosing fluid deficiencies.

Enhanced recovery after surgery guidelines suggest that dynamic monitoring indicators may replace the CVP as an indicator of fluid responsiveness [25, 26]. Real-time monitoring of the oxygen demand–supply imbalance associated with hepatectomy enables an early detection and treatment of abnormalities and prevents perioperative complications. Previous studies have demonstrated that patients who underwent FTS-monitored anesthetic management had a good postoperative course [6, 27, 28]. The SVV, an FTS-measured indicator of fluid responsiveness, is useful for the perioperative management of patients undergoing highly invasive surgery [6, 29]. Moreover, the SVV is better than the CVP as a predictor of fluid responsiveness during hepatectomy [27]. An intraoperative mean SVV ≥ 13.6 has been reported to increase postoperative total bilirubin levels [5]. However, our study showed no relationship between the SVV and CCI score. Although the SVV is an index of fluid responsiveness, it does not assess tissue oxygenation. The oxygen demand–supply balance may be undisturbed even when the SVV is high. In addition, the SVV cannot be accurately assessed in patients with arrhythmias or in those undergoing laparoscopic surgery [11, 30].

The FTS can also measure the ScvO_2_, which is an indicator of oxygen demand–supply balance. Oxygen deprivation can lead to mitochondrial dysfunction-induced organ damage [31], which reduces resistance to postoperative stress, thereby increasing the occurrence of postoperative complications. Patients with low intraoperative ScvO_2_ values are more predisposed to complications after high-risk surgical procedures [32]. During hepatectomy, ischemia–reperfusion injury caused by the Pringle maneuver alters the balance of hepatic oxygen supply [1, 5]. The results of FTS, including ScvO2, are influenced by vascular compliance and peripheral vascular resistance. Vascular compliance is estimated from age, sex, height, and weight [11]. Above mentioned factors were not significantly different between two groups in this study. The optimal cutoff ScvO_2_ value for predicting postoperative complications differs between healthy patients and those with trauma, severe sepsis, and heart failure [33, 34]. It is difficult to determine the standard ScvO_2_ value for all patients; nevertheless, the postoperative course can be improved via intraoperative ΔScvO_2_ suppression. More detailed studies are needed on the factors and mechanisms involved in ScvO2 fluctuations.

Furthermore, we found a relationship between the CCI score and CLR in univariate analysis. Preoperative inflammatory biomarkers have been shown to be associated with the incidence of postoperative complications after esophagectomy [35, 36]. The postoperative course is affected by preoperative lymphocyte count and C-reactive protein levels, which are involved in immune and inflammatory reactions, respectively. CLR is thought to predict the postoperative status better than other inflammatory biomarkers. If the number of cases increases, preoperative CLR may become effective predictor of postoperative complications in hepatectomy.

This study has some potential limitations. First, the anesthetic management method was not standardized; it was selected at the discretion of each anesthesiologist. Therefore, the method used by the anesthesiologist may have influenced intraoperative indicators. Second, the study was a retrospective, single-center cohort with a small sample size. This may cause of the lack of significant differences in ΔScvo2 and individual complications, although a trend was observed for pleural effusions and DGE. Therefore, the study findings should be verified via large-scale, multicenter randomized controlled trials.

In conclusion, ScvO_2_ monitoring using the FTS can be used as an alternative to CVP monitoring and lactate level measurement to predict the risk of postoperative complications. Given the association between change in ScvO_2_ and postoperative complications, minimizing the change in ScvO_2_ is a potential strategy for decreasing the risk of postoperative complications after hepatectomy.

### Supplementary Information

**Additional file 1.**

## Data Availability

The datasets generated and/or analyzed during the current study are not publicly available due to institutional policies but are available from the corresponding author on reasonable request.

## Abbreviations

CVP: Central venous pressure
FTS: Flo Trac system
ScvO2: Central venous oxygen saturation
ΔScvO_2_: Central venous oxygen saturation fluctuation
SVV: Stroke volume variation
ΔSVV: Stroke volume variation fluctuation
CCI: Comprehensive complication index
ORs: Odds ratios
CIs: Confidence intervals
CLR: C-reactive protein-to-lymphocyte ratio
