# Supplementary material for: Prediction of postoperative complications after hepatectomy with dynamic monitoring of central venous oxygen saturation
Source: BMC Surg. 2023 Nov 14;23:343. doi: 10.1186/s12893-023-02238-6 (PMC10644466; doi:10.1186/s12893-023-02238-6)
Supplement: Supplementary file 1 — Additional file 1. [file 12893_2023_2238_MOESM1_ESM.zip › AdditionalFile BMC Surgery.docx]

**Additional file 1. Patient data stratified by average SVV**

|  | Low average SVV  (SVV <13.6)  (n=45) | High average SVV  (SVV ≥13.6)  (n=8) | *p* |
| --- | --- | --- | --- |
| **Preoperative factors** |  |  |  |
| Age | 70 (63–77) | 68 (60–70) | 0.171 |
| Sex (male/female) | 36: 9 | 4: 4 | 0.090 |
| BMI | 22.3 (20.5–24.7) | 22.7 (20.4–24.8) | 0.798 |
| ASA (1: 2: 3) | 0: 41: 4 | 1: 7: 0 | 0.242 |
| Hypertension (%) | 13/45 (28.9) | 2/8 (25.0) | 0.596 |
| Hyperlipidemia (%) | 3/45 (6.7) | 0/8 (0) | 0.606 |
| Diabetes (%) | 10/45 (22.2) | 2/8 (25.0) | 0.588 |
| Primary disease (HCC: others) | 25: 20 | 4: 4 | 0.534 |
| PTPE (%) | 4/45 (8.9) | 0/8 (0) | 0.509 |
| History of hepatitis virus (HBV: HCV: no) | 4: 5: 36 | 2: 2: 4 | 0.090 |
| Child-Pugh classification (A: B: C) | 44: 1: 0 | 8: 0: 0 | 0.849 |
| Liver damage classification (A: B: C) | 38: 7: 0 | 8: 0: 0 | 0.294 |
| White blood cell (/μL) | 5360 (4460–6560) | 4915 (4113–5603) | 0.286 |
| Platelet (×10^4^/μL) | 19.5 (15.5–23.8) | 17.4 (16.0–22.8) | 0.533 |
| Prothrombin activity (%) | 102 (96–115) | 106 (92–114) | 0.971 |
| Aspartate transaminase (IU/L) | 25 (21–40) | 28 (26–32) | 0.617 |
| Alanine transaminase (IU/L) | 20 (15–31) | 26 (19–36) | 0.333 |
| Total bilirubin (mg/dL) | 0.7 (0.6–1.0) | 1.2 (0.9–1.5) | **0.019** |
| Alkaline phosphatase (IU/L) | 239 (199–310) | 202 (159–205) | **0.021** |
| γ-Glutamyltranspeptidase (IU/L) | 55 (27–92) | 27 (22–35) | **0.026** |
| Albumin (g/dL) | 4.2 (3.8–4.4) | 4.4 (4.3–4.5) | 0.116 |
| Cholinesterase (IU/L) | 278 (232–312) | 282 (266–351) | 0.333 |
| Total cholesterol (mg/dL) | 188 (161–213) | 197 (181–228) | 0.309 |
| C-reactive protein (mg/dL) | 0.13 (0.05–0.37) | 0.05 (0.03–0.09) | **0.028** |
| HbA1c (%) | 5.9 (5.5–6.3) | 6.1 (5.6–6.3) | 0.742 |
| ICG R15 (%) | 16.2 (12.0–23.1) | 14.1 (11.2–17.1) | 0.389 |
| NLR | 3.05 (2.04–4.50) | 2.99 (2.30–3.45) | 0.517 |
| PLR | 169.9 (116.0–265.7) | 145.0 (130.3–197.8) | 0.652 |
| LMR | 3.22 (2.45–4.45) | 3.87 (3.42–4.78) | 0.268 |
| CLR | 9.87 (4.88–32.42) | 4.49 (2.68–8.36) | **0.034** |
| CAR | 0.030 (0.011–0.097) | 0.010 (0.007–0.021) | **0.028** |
| **Intraoperative factors** |  |  |  |
| Operative method  (minor: major) | 38: 7 | 8: 0 | 0.294 |
| laparotomy: laparoscopy | 26: 19 | 2: 6 | 0.092 |
| Operation time (min) | 317 (261–388) | 272 (239–293) | 0.075 |
| Intraoperative bleeding (mL) | 275 (85–542) | 33 (13–86) | **0.005** |
| Urine volume (mL) | 279 (110–406) | 186 (118–270) | 0.385 |
| Transfusion (%) | 5/45 (11.1) | 0/8 (0) | 0.426 |
| Crystalloid fluid volume (mL) | 2100 (1700–2720) | 1745 (1573–1825) | 0.065 |
| Intraoperative in-out balance (mL/kg/h) | 6.75 (5.59–8.11) | 6.52 (5.17–7.26) | 0.600 |
| Total Pringle maneuver time (min) | 90 (60–110) | 78 (60–96) | 0.481 |
| Hepatectomy time (min) | 117 (82–177) | 110 (93–147) | 0.732 |
| **Postoperative factors** |  |  |  |
| Max white blood cell (/μL) | 10190 (8350–12130) | 7790 (6878–11713) | 0.286 |
| Min platelet (×10^4^/μL) | 14.3 (9.4–17.0) | 13.5 (11.5–15.5) | 0.932 |
| Min prothrombin activity (%) | 69 (60–80) | 78 (64–97) | 0.321 |
| Max aspartate transaminase (IU/L) | 241 (164–586) | 183 (162–286) | 0.358 |
| Max alanine transaminase (IU/L) | 245 (137–467) | 211 (170–332) | 0.913 |
| Max total bilirubin (mg/dL) | 1.2 (1.0–1.9) | 1.5 (1.3–1.8) | 0.333 |
| Min Albumin (g/dL) | 2.9 (2.7–3.2) | 3.3 (3.2–3.5) | **0.010** |
| Min Cholinesterase (IU/L) | 167 (142–197) | 237 (183–268) | **0.018** |
| Max C-reactive protein (mg/dL) | 9.47 (6.34–12.39) | 7.29 (5.97–9.01) | 0.214 |
| Complications of CDC grade IIIa or higher (yes: no) | 2: 43 | 0: 8 | 0.718 |
| CCI | 8.7 (0–20.9) | 0 (0–3.1) | 0.116 |
| Postoperative length of stay (day) | 10 (8–13) | 7 (7–9) | **0.032** |

Continuous data are presented as median (interquartile range), whereas categorical data are shown as number of patients. Significant p-values are in boldface.

SVV, stroke volume variation; BMI, body mass index; ASA, American Society of Anesthesiologists; HCC, hepatocellular carcinoma; PTPE, Percutaneous transhepatic portal vein embolization; HCV, hepatitis C virus; HBV, hepatitis B virus; HbA1c, Hemoglobin A1c; ICGR15, indocyanine green retention rate at 15 min; NLR, neutrophil-to-lymphocyte ratio; PLR, platelet-to-lymphocyte ratio; LMR, lymphocyte-to-monocyte ratio; CLR, C-reactive protein-to-lymphocyte ratio; CAR, C-reactive protein-to-albumin ratio; CDC, Clavien-Dindo classification; CCI, Comprehensive complication
