# Supplementary material for: Prediction of postoperative complications after hepatectomy with dynamic monitoring of central venous oxygen saturation
Source: BMC Surg. 2023 Nov 14;23:343. doi: 10.1186/s12893-023-02238-6 (PMC10644466; doi:10.1186/s12893-023-02238-6)
Supplement: Supplementary file 1 — Additional file 1. [file 12893_2023_2238_MOESM1_ESM.zip › Supplementary material legends BMC Surgery.docx]

**Supplementary Material**

**File name: Additional file 1**

File format: .docx

Title of data: Patient data stratified by average SVV

Description of data: Additional file 1 shows the patient background, preoperative treatment, preoperative blood test, intraoperative factors, postoperative blood test, and postoperative course stratified by average SVV.
